# Immediate Effects of Footwear Design on In‐Shoe Plantar Pressures, Impact Forces and Comfort in Women With Plantar Heel Pain

**DOI:** 10.1002/jfa2.70055

**Published:** 2025-06-13

**Authors:** Melinda M. Franettovich Smith, Wolbert van den Hoorn, Adele van den Hoek, Graham Kerr, Sheree E. Hurn

**Affiliations:** ^1^ School of Clinical Sciences Faculty of Health Queensland University of Technology Brisbane Australia; ^2^ School of Health and Rehabilitation Sciences Faculty of Health and Behavioural Sciences The University of Queensland Brisbane Australia; ^3^ School of Exercise and Nutrition Sciences Faculty of Health Queensland University of Technology Brisbane Australia; ^4^ Australian Centre for Health Services Innovation and Centre for Healthcare Transformation School of Public Health and Social Work Faculty of Health Queensland University of Technology Brisbane Australia

**Keywords:** foot orthoses, shoe insert, shoes

## Abstract

**Background:**

Footwear is often recommended in the management of plantar heel pain (PHP), theoretically to reduce tissue stress during standing and walking; however, limited data exist to guide footwear design and recommendations.

**Methods:**

Plantar pressures, impact forces and comfort during walking were recorded in 29 women with PHP (mean age 47 ± 12 years) in six randomised shoe and insert conditions. A test shoe (polyurethane outsole, 14‐mm heel‐toe pitch) was compared to a control shoe (rubber outsole, 4‐mm heel‐toe pitch), and within the test shoe, five different insoles that varied by material, density and arch contouring were also compared.

**Results:**

The test shoe reduced heel peak pressure (15%, *p* < 0.01) and reduced the loading rate but not the peak magnitude of the vertical ground reaction force (average loading rate reduction: 7%, *p* < 0.01; maximum loading rate: 29%, *p* < 0.01) and was more comfortable (47%–67%, *p* < 0.01) compared to the control shoe. Within the test shoe, dual‐density inserts with arch contouring showed lower heel peak pressure compared to a lightweight flat insert (11%–12%, *p* < 0.03). The insert with the firmest material and higher arch contouring showed higher midfoot peak pressure (16%–21%, *p* < 0.01) compared to other inserts. Forefoot peak pressure did not differ between shoe or insert conditions (*p* > 0.05). There were no differences in impact forces or comfort between the different inserts within the test shoe (*p* > 0.05).

**Conclusion:**

Findings suggest that shoe and insert properties are both important and provide data to guide footwear design and management recommendations for PHP.

## 1 Introduction

Plantar heel pain (PHP) is among the most common symptoms reported by people with foot pain who seek medical treatment [1, 2]. The condition has a detrimental impact on health‐related quality of life and is associated with limited physical and social capacity [3, 4, 5]. Imaging studies have demonstrated that many structures of the plantar heel are affected in PHP [6], and the heel fat pad is less effective at dissipating energy in affected individuals compared to those without PHP [7]. One typical clinical feature of PHP is that symptoms are aggravated by weight‐bearing activities [8]. In some occupational settings, increased time standing on hard surfaces and walking have been associated with PHP [9, 10]. Shoes and shoe inserts that have the potential to alter weight‐bearing loads on structures of the heel are therefore considered an important component of management [8, 11].

In PHP populations, various shoe inserts (contoured orthoses, heel cups and heel pads) have been shown to reduce heel peak pressures [12, 13, 14, 15]; however, effects of shoe design and material properties have not yet been investigated in PHP. Findings in asymptomatic populations report reduced plantar pressures in shod walking compared to barefoot [16, 17], with more compressible (softer) materials redistributing loads effectively [18, 19] and softer‐soled shoes producing lower peak pressures than harder‐soled shoes [20]. Further investigation of shoe design and materials in PHP populations is therefore warranted.

Although pressure‐relieving properties of shoes and shoe inserts have been evaluated, their shock‐attenuating effects have not been investigated in PHP, and studies in asymptomatic adults report conflicting findings on shoe inserts [21, 22, 23, 24, 25]. Studies of impact forces during walking and running in asymptomatic individuals report varied effects of shoes compared to barefoot [16, 17] and minimal differences between different military and training footwear designs [26]. Investigation of the potential effect of shoes and shoe inserts on impact forces during walking in people with PHP will improve understanding of the role of footwear in clinical management.

The existing literature on pressure‐relieving and shock‐attenuating effects of shoes and shoe inserts largely focuses on athletic footwear [12, 13, 16, 17, 21] and military footwear [26], and in some cases, footwear features are not clearly described [14, 15, 25]. Many occupations involving long hours of weight‐bearing (e.g., nursing, teaching, retail, hospitality) require people to wear nonathletic styles of footwear. There is currently a lack of evidence investigating the pressure‐relieving and shock‐attenuating properties of nonathletic ‘everyday’ footwear and inserts that may fit in such footwear. Additionally, there is limited understanding of how pressure‐relieving and shock‐attenuating properties of footwear influence comfort.

Experts recommend that individuals with PHP should be advised to select comfortable footwear [11]. In a small sample with PHP, Ho et al. (2022) reported that walking in a canvas sneaker with a contoured custom orthotic was rated more comfortable than without an orthotic [27]. This is supported by systematic review evidence from varied populations that adding an insole improved footwear comfort [28]. The existing literature suggests that softer, more flexible insoles are perceived as more comfortable, but insole preference is also influenced by occupation [28]. By comparison, shoe sole hardness was reported to have minimal influence on comfort [20]. The influence of insole contouring is less clear, with conflicting evidence as to whether flat or contoured insoles are perceived as more comfortable [28]. Comfort of shoes and shoe inserts is an important consideration because it influences adherence [29].

To the authors' knowledge, no study has concurrently evaluated the pressure‐relieving properties, impact forces and comfort of shoes and shoe inserts in individuals with PHP. Improving our understanding of footwear features that provide cushioning and comfort for individuals with PHP may be an important first step to identify a footwear intervention to investigate in a clinical trial. The aim of this study was to investigate the effect of both shoe and shoe insert designs on in‐shoe plantar pressures, vertical ground reaction force and underfoot comfort in individuals with PHP.

## 2 Materials and Methods

This study was approved by the Institutional Human Research Ethics Committee (#4889) and participants provided written informed consent. Funding was provided by a government and industry partnership scheme (Innovation Connections ICG001752). The industry partner manufactures women's footwear; hence, women were recruited to participate in this study. The industry partner was not involved in data collection or analysis.

Twenty‐nine women with PHP participated in this study. Participants were recruited via advertisements in local physiotherapy and podiatry clinics, the university community and paid Facebook advertisements. Eligible women were aged 18 years or older and reported symptoms consistent with PHP in one or both feet for a minimum of 4 weeks. Inclusion criteria also included pain aggravated by walking on most days in the last month (rated at least three out of 10 on a numerical rating scale), pain with palpation of the plantar heel and shoe size women's US/AUS six to eleven. Women were excluded if they reported a mobility or cognitive impairment, neurological condition affecting walking gait, injury or surgery of the lower back or lower limb in the preceding six months, lower limb musculoskeletal pain in areas other than the heel that may affect walking gait, systemic inflammatory disease, peripheral sensory neuropathy or regular use of foot orthoses in the last 12 months. Participant characteristics are displayed in Table 1. Sample size calculations indicated that 26 participants provided a 90% probability of detecting a moderate difference between conditions (*f* = 0.25) with the alpha level set at 0.05 [30].

**TABLE 1:** Characteristics of the women who participated in this study.

Characteristic	Mean (SD)	Range
Age (years)	47 (12)	24–73
Height (m)	1.65 (0.06)	1.51–1.73
Weight (kg)	83.4 (13.5)	57.5–103.1
Body mass index (kg/m^2^)	30.7 (5.0)	20.5–39.3
Time on feet (hours/day)	6.2 (2.8)	1.5–12.0
Foot posture index (−12 to +12)	+3.5 (3.7)	−4 –+10
Manchester‐Oxford Foot Questionnaire (%, 100 = greater severity)		
Index (single score)	54.1 (15.2)	23.4–85.9
Pain	60.7 (2.5)	25.0–85.0
Standing/walking function	48.4 (19.3)	25.0–92.9
Social	42.9 (21.6)	0.0–87.5

Participants attended one data collection session where they walked in six conditions across a 10‐m walkway. Each participant was fitted for shoe and insert size and provided a new pair of socks (bamboo ankle sock, FRANKIE4). The order of conditions was randomised to minimise potential sequencing effects. Data were recorded for the painful limb or most painful limb where symptoms were bilateral. To describe participant characteristics we recorded age, weight, height, average time spent standing each day, foot posture index [31] and foot pain and disability (Manchester‐Oxford Foot Questionnaire) [32].

**FIGURE 1:**
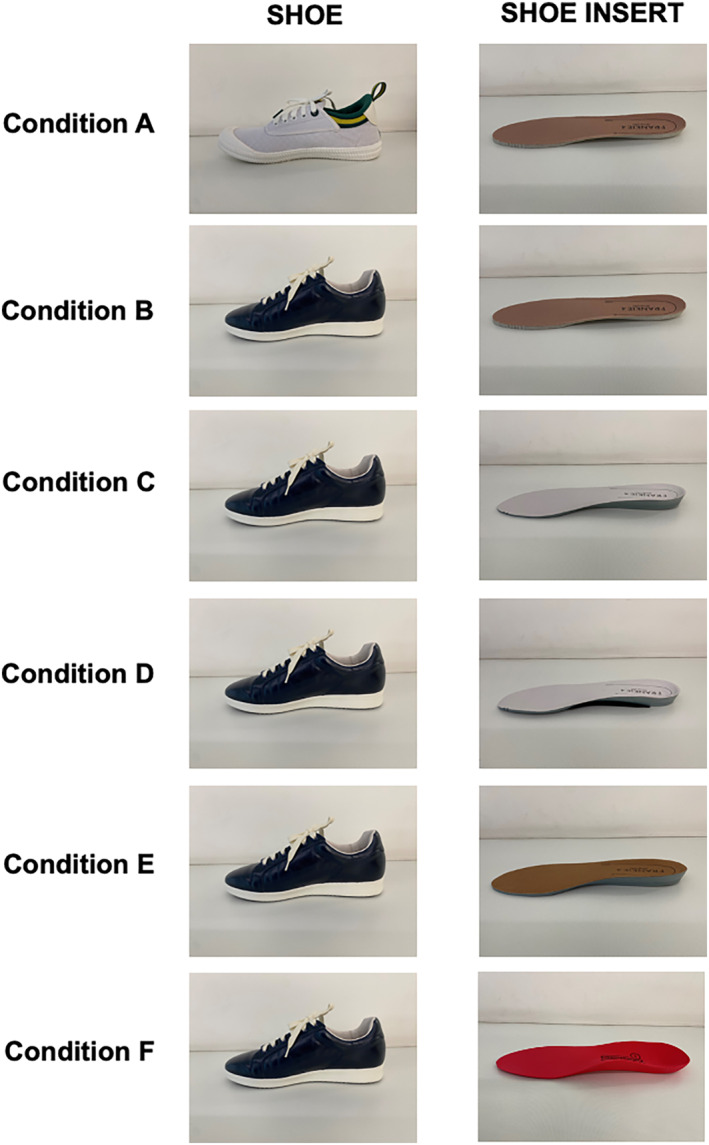
Shoe and insert conditions. The test shoe was a lace‐up sneaker with leather upper, 14‐mm heel‐toe pitch and dual‐density moulded polyurethane outsole (Jackie sneaker, FRANKIE4, Australia). The control shoe was an athletic lace‐up sneaker with canvas upper, 4‐mm heel‐toe pitch and rubber outsole (Dunlop Volley, Pacific Dunlop Ltd., Melbourne, Australia). Condition A: Control shoe (Dunlop Volley) with a flat low‐density ethylene vinyl acetate foam insert (FRANKIE4 Flat + Lite Footbed). Condition B: Test shoe (Jackie sneaker, FRANKIE4) with a flat low‐density ethylene vinyl acetate foam insert (FRANKIE4 Flat + Lite Footbed). Condition C: Test shoe (Jackie sneaker, FRANKIE4) with a moulded contoured dual‐density insert (FRANKIE4 Sole Hero Footbed). Condition D: Test shoe (Jackie sneaker, FRANKIE4) with a moulded contoured dual‐density insert (FRANKIE4 Sole Hero Footbed) with a moulded contoured thermoplastic rubber piece at midfoot (FRANKIE4 Arch Peace). Condition E: Test shoe (Jackie sneaker, FRANKIE4) with a moulded contoured bio‐derived polyurethane (PU) dual‐density insert (FRANKIE4 Sole Hero Bio PU Footbed). Condition F: Test shoe (Jackie sneaker, FRANKIE4) with a moulded contoured polyethylene foam insert (Formthotics).

To investigate the effect of shoe outsole design, we compared a test shoe (Jackie sneaker, FRANKIE4, Australia; lace‐up sneaker with 14‐mm heel‐toe pitch and dual‐density moulded polyurethane outsole) with design features consistent with expert recommendations for PHP [11] (i.e., supportive, incorporating some heel‐toe pitch) to a control shoe lacking these features (Dunlop Volley, Pacific Dunlop Ltd., Melbourne, Australia; lace‐up sneaker with a 4‐mm heel‐toe pitch and rubber outsole). For this comparison, the same flat low‐density ethylene vinyl acetate foam insert was used (FRANKIE4 Flat + Lite Footbed); see Conditions A and B, Figure 1. Control and test shoes were re‐used throughout this study. To investigate the effect of insert design, within the test shoe we compared five inserts that varied in material and contouring; see Conditions B–F, Figure 1. A new shoe insert was used for each participant for Conditions A–E and every second participant for Condition F. Condition F insert was trimmed and additionally heat‐moulded to fit inside the shoe (other conditions did not require heat moulding). Participants were aware that different shoe/insert conditions were being assessed but were not informed of specific features. The six conditions tested were as follows (Figure 1):

- Control shoe with a flat low‐density ethylene vinyl acetate foam insert (FRANKIE4 Flat + Lite Footbed).
- Test shoe with a flat low‐density ethylene vinyl acetate foam insert (FRANKIE4 Flat + Lite Footbed).
- Test shoe with a moulded contoured dual‐density insert (FRANKIE4 Sole Hero Footbed) to investigate the effect of this insert material.
- Test shoe with a moulded contoured dual‐density insert (FRANKIE4 Sole Hero Footbed) with a moulded contoured thermoplastic rubber piece at midfoot (FRANKIE4 Arch Peace) to investigate the effect of increased contouring.
- Test shoe with a moulded contoured bio‐derived polyurethane (PU) dual‐density insert (FRANKIE4 Sole Hero Bio PU Footbed) to investigate the effect of this insert material.
- Test shoe with a moulded contoured polyethylene foam insert (Formthotics) as the industry standard comparator.

The Novel Pedar system (Novel GmbH, Munich, Germany) collected in‐shoe plantar pressure data during walking trials. For each condition, the Pedar insole was placed atop each insert and calibrated in a non‐weight‐bearing position prior to a two‐minute acclimatisation walk. A force plate (AMTI, OR6‐6, Watertown, MA, USA) embedded in the floor measured vertical ground reaction force (vGRF). Force plate data were digitised using a Vicon MX Giganet system (Vicon, Yarnton, Oxford, UK) at 1000 samples/s. Reflective markers (14 mm) were placed on the shoe at the heel and second toe to enhance visualisation confirming force plate foot strike and on the participant's back at the level of the sacrum to assess variability in walking speed. 3D positions of reflective markers were recorded with the force plate data at 100 samples/s using a 12‐camera Vicon system (Vantage V5, Vicon, Yarnton, Oxford, UK). Participants walked at a self‐selected comfortable speed until five successful trials (i.e., consistent speed, force plate strike with test limb) were recorded. Timing gates (Freelap SA, Switzerland) were used to monitor walking speed in real time with trials excluded if they varied more than 5% from the original self‐selected walking time. At the completion of walking trials in each condition, participants used a 100‐mm visual analogue scale to rate their perceived comfort underfoot (anchored by ‘not comfortable at all’ and ‘most comfortable imaginable’) at the heel, midfoot and forefoot regions as well as overall (i.e., under the whole foot) [33]. To monitor symptoms across the session, participants completed a 100‐mm visual analogue scale to rate perceived PHP intensity while walking (anchored by ‘no pain’ and ‘worst pain imaginable’). Participants were provided a minimum five‐minute rest period between each condition.

Data processing was performed by investigators blinded to condition. Using the Novel Pedar‐x Expert analysis program (version 28.3.8.6, Novel GmbH, Munich, Germany), the middle four steps from each trial were selected for analysis. A mask was applied to each footprint, corresponding to the heel, midfoot and forefoot regions [34]. Peak pressure and contact area were extracted for each stance phase, and the average for each condition was determined from 20 steps (four steps × five trials per condition).

Force plate data were low‐pass filtered using a bi‐directional second‐order low‐pass Butterworth filter with a cut‐off frequency at 100 Hz [35]. The stance phase period was detected using a vertical force threshold of 1 N. Vertical force plate data were normalised to body weight using the mean of vertical force during a ∼10 s stationary standing period. Within each stance phase, peak magnitude was identified as the first maximum, average loading rate was calculated from heel strike till peak magnitude, and peak loading rate was calculated as the maximum rate of change during the same time period. 3D positional data were low‐pass filtered using a second‐order Butterworth filter at 5 Hz [36]. To verify that consistent walking speed was achieved across conditions, walking speed was calculated in post‐processing as the mean of the time‐differentiated sacral marker position in the X‐direction across the complete stride cycle prior to force plate heel strike. Heel strike prior to force plate heel strike was determined as the local vertical minimum of the heel marker [37]. Step length was determined as the difference in X position of the heel marker at force plate strike and the X position of the contralateral heel during the prior heel strike.

For plantar pressure and force plate data, the coefficient of variation of stance time for included steps was less than 10%, which was deemed acceptable. Walking speed for each trial across conditions was inspected to ensure all conditions were within ±5% variation.

The Statistical Package for Social Sciences (version 27, SPSS Inc, Chicago, IL) was used for data analysis. Data were explored for normality using histograms and skewness/kurtosis statistics (values above +3 or below −3 were considered a good indication that data were not normally distributed) [38]. Between‐condition differences in peak pressure, contact area, peak vGRF, average and maximum loading rate of the vGRF and comfort were determined using a linear mixed model and post hoc tests where appropriate. To investigate the effect of shoe outsole design, the comparison of Conditions A and B was of interest, and to investigate the effect of insert design, Condition B through Condition F were compared. For the contact area at the heel/midfoot/forefoot, bootstrapping (conducted with 5000 resamples) was conducted, as the transformation of data was not successful in meeting assumptions of normal distribution. To ensure gait consistency, a linear mixed model was used to examine between‐condition differences in speed, stance time and step length. To monitor the stability of pain across conditions, a linear mixed model was used to examine differences in pain across the order of conditions (A–F). For all analyses, differences between conditions were considered significant if *p* < 0.05. For each linear mixed model, participants' identification numbers were entered as random intercepts and condition was entered as a fixed factor.

## 3 Results

Speed, stance time and step length were not different between conditions (Table 2). Pain did not differ across the order of the six conditions (*p* = 0.98; i.e., pain was stable through the data collection session).

**TABLE 2:** Group mean and standard deviation for speed, stance time, peak plantar pressures, contact area, vertical ground reaction force (GRF) and comfort for each condition.

Variable	Condition A	Condition B	Condition C	Condition D	Condition E	Condition F	*p*‐value
Speed (m/s)	1.32 (0.17)	1.32 (0.17)	1.33 (0.17)	1.33 (0.17)	1.32 (0.18)	1.32 (0.17)	0.99
Stance time (s)	0.684 (0.050)	0.695 (0.050)	0.694 (0.051)	0.692 (0.050)	0.696 (0.053)	0.694 (0.050)	0.34
Step length (m)	0.70 (0.07)	0.71 (0.07)	0.72 (0.07)	0.71 (0.07)	0.71 (0.07)	0.71 (0.07)	0.54
Peak plantar pressure (kPa) by region							
Heel	301.5 (65.0)^b^, ^c^	256.8 (45.0)^a^	233.0 (44.2)	225.0 (45.2)^b^, ^c^	229.7 (45.9)^b^, ^c^	233.4 (37.9)^c^	< 0.01
Midfoot	118.0 (33.4)^c^	122.4 (28.3)	120.4 (25.4)	128.0 (25.4)^c^	117.6 (31.3)^c^	142.4 (36.7)^b^, ^c^	0.02
Forefoot	356.6 (76.7)^c^	345.0 (55.0)	355.5 (57.2)	352.8 (52.1)^c^	357.1 (62.0)^c^	393.0 (61.2)^c^	0.07
Contact area (mm^2^) by region							
Heel	37.3 (2.2)	37.5 (2.1)	37.3 (2.3)	37.5 (2.1)	37.2 (2.6)	37.4 (2.2)	0.99
Midfoot	36.8 (3.8)	36.7 (4.6)	38.1 (3.7)	38.2 (5.0)	37.4 (6.1)	38.9 (4.9)	0.43
Forefoot	64.8 (4.3)	64.7 (4.4)	64.3 (4.5)	64.8 (3.8)	63.8 (4.3)	64.4 (3.6)	0.93
vGRF (normalised to body weight)							
Peak magnitude of force (BW)	1.10 (0.09)^c^	1.09 (0.08)^c^	1.11 (0.09)^d^	1.11 (0.08)	1.11 (0.08)^c^	1.10 (0.09)^d^	0.44
Average loading rate (BW.s)	7.90 (1.87)^b^, ^c^	7.34 (1.61)^a^, ^c^	7.33 (1.73)^d^	7.40 (1.76)	7.30 (1.84)^c^	7.28 (1.74)^d^	0.03
Maximum loading rate (BW.s)	22.24 (10.19)^b^, ^c^	15.82 (4.56)^a^, ^c^	16.23 (5.49)^d^	16.44 (6.22)	15.69 (4.97)^c^	16.22 (4.96)^d^	< 0.01
Comfort (based on a 100‐mm visual analogue scale)							
Overall (mm)	36.2 (24.7)^b^	59.4 (22.4)^a^	63.3 (19.6)	62.9 (24.7)	55.21 (22.6)	55.2 (23.9)	< 0.01
Heel (mm)	33.6 (22.3)^b^	56.0 (21.5)^a^	60.3 (22.1)	63.0 (22.5)	56.4 (21.1)	52.2 (23.3)	< 0.01
Midfoot (mm)	36.8 (23.7)^b^	60.3 (21.8)^a^	65.3 (22.6)	62.5 (25.8)	55.8 (25.0)	55.5 (29.3)	< 0.01
Forefoot (mm)	45.4 (29.9)^b^	66.6 (21.5)^a^	67.4 (21.8)	66.4 (23.6)	63.6 (22.5)	61.1 (25.2)	< 0.01

*Note:* Condition A: Control shoe (Dunlop Volley) with a flat low‐density ethylene vinyl acetate foam insert (FRANKIE4 Flat + Lite Footbed). Condition B: Test shoe (Jackie sneaker, FRANKIE4) with a flat low‐density ethylene vinyl acetate foam insert (FRANKIE4 Flat + Lite Footbed). Condition C: Test shoe (Jackie sneaker, FRANKIE4) with a moulded contoured dual‐density insert (FRANKIE4 Sole Hero Footbed). Condition D: Test shoe (Jackie sneaker, FRANKIE4) with a moulded contoured dual‐density insert (FRANKIE4 Sole Hero Footbed) with a moulded contoured thermoplastic rubber piece at midfoot (FRANKIE4 Arch Peace). Condition E: Test shoe (Jackie sneaker, FRANKIE4) with a moulded contoured bio‐derived polyurethane (PU) dual‐density insert (FRANKIE4 Sole Hero Bio PU Footbed). Condition F: Test shoe (Jackie sneaker, FRANKIE4) with a moulded contoured polyethylene foam insert (Formthotics). The test shoe was a lace‐up sneaker with leather upper, 14‐mm heel‐toe pitch and dual‐density moulded polyurethane outsole (Jackie sneaker, FRANKIE4, Australia). The control shoe was an athletic lace‐up sneaker with canvas upper, 4‐mm heel‐toe pitch and rubber outsole (Dunlop Volley, Pacific Dunlop Ltd., Melbourne, Australia). *p*‐value based on a linear mixed model.

Abbreviation: BW, body weight.

^a^ Different to Condition A in post‐hoc comparisons (*p* < 0.05).

^b^ Different to condition B in post‐hoc comparisons (*p* < 0.05).

^c^ Missing data for 1 participant.

^d^ Missing data for 2 participants (missing data for peak pressures was due to signal loss at data collection. Missing data for vertical GRF was due to the loss of sacral reflective marker and inability to verify walking speed).

**FIGURE 2:**
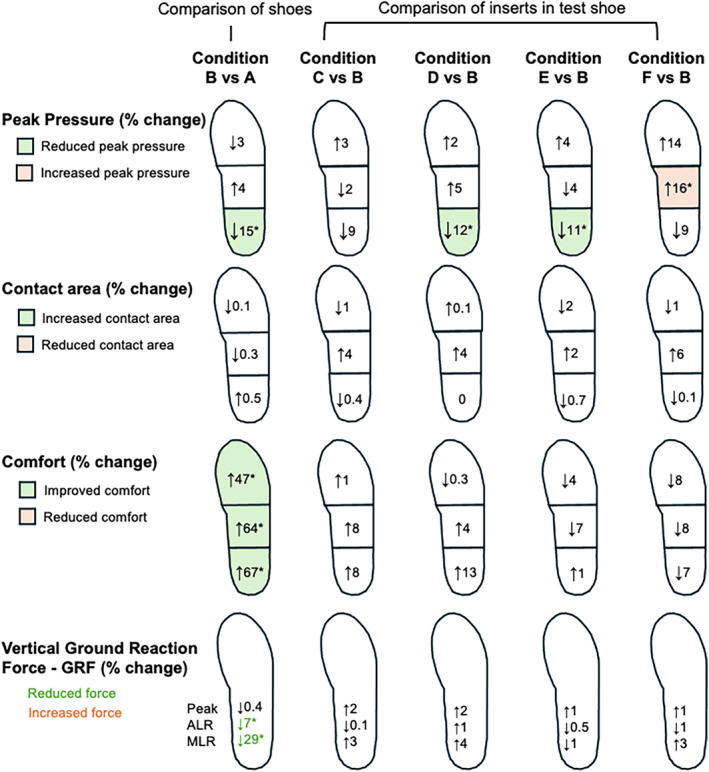
Percentage change in peak pressure, contact area, comfort and vertical GRF for shoe (Condition A compared to B) and insert (Condition C/D/E/F compared to B) comparisons. Significant (*p* < 0.05) differences marked with an asterisk (*) with colour indicating the direction of difference (green for reduced, orange for increased). ALR = average loading rate; MLR = maximum loading rate.

Heel peak pressure was lower (mean difference: 45 kPa, 15%, *p* < 0.01) in the test shoe (Condition B) compared to the control shoe (Condition A) (Table 2, Figure 2). Within the test shoe, Conditions D and E reduced heel peak pressure when compared to Condition B (31.8 kPa, 12%, *p* = 0.01; 27.1 kPa, 11%, *p* = 0.03). Midfoot peak pressure was higher in Condition F compared to Condition B (20.0 kPa, 16%, *p* = 0.01). Forefoot peak pressure was not different between shoe or insert conditions (< 0.4–36 kPa, < 0.1%–10%, *p* = 0.07). Contact area at the heel, midfoot and forefoot was not different between conditions (< 2.1 cm^2^, < 6%, *p* = 1.0, 0.43 and 0.93, respectively) (Table 2, Figure 2).

Peak magnitude of the normalised vGRF was not different between conditions (*p* = 0.44). Average and maximum loading rates were lower (average loading rate: 0.56 BW/s, 7%, *p* < 0.01; maximum loading rate: 6.42 BW/s, 29%, *p* < 0.01) in the test shoe (Condition B) compared to the control shoe (Condition A) (Table 2, Figure 2). Within the test shoe, there were no differences observed between insert conditions (*p* > 0.33).

Perceived underfoot comfort overall and at the heel, midfoot and forefoot was higher in the test shoe (Condition B) compared to the control shoe (Condition A) (Table 2, Figure 2). Improvements in comfort were 23.1 mm (64%, *p* < 0.01) for overall comfort, 22.4 mm (67%, *p* < 0.01) at the heel, 23.5 (64%, *p* < 0.01) at the arch and 21.2 (47%, *p* < 0.01) at the forefoot. Within the test shoe, there were no differences in comfort observed between insert conditions (*p* > 0.07).

## 4 Discussion

Our findings demonstrate the importance of both shoe and shoe insert design. In women with PHP, a nonathletic ‘everyday’ sneaker with design features consistent with recommendations for PHP reduced heel peak pressure and loading rate of the vGRF during walking and was rated as more comfortable than a shoe without these features. Within the test shoe, some inserts influenced peak plantar pressures, but there were no differences in impact forces or comfort between the various inserts.

Plantar pressures at the heel were influenced by both shoe and shoe insert design, highlighting the potential increased efficacy of inserts in combination with recommended footwear. The test shoe with a flat low‐density ethylene vinyl acetate foam insert (Condition B) reduced heel peak pressure compared to the control shoe with the same insert (Condition A). Differences in shoe outsole design included softer/more compressible material (polyurethane vs. rubber) and greater heel‐toe pitch (14 vs. 4 mm). This finding is supported by findings in asymptomatic individuals where plantar pressures were higher as sole hardness increased [20]. Within the test shoe, dual‐density inserts with arch contouring (Conditions D and E) more effectively offloaded heel peak pressure compared to a flat low‐density ethylene vinyl acetate insert (Condition B). Both contouring and material compressibility have been shown to influence peak plantar pressures at the heel in asymptomatic individuals [18]. These inserts effectively reduced heel peak pressure without significantly increasing pressure at the midfoot or forefoot. The insert with the firmest material and higher arch contouring (Condition F) increased midfoot peak pressures. Our observation of increased midfoot peak pressure with this insert is similar to that reported by a previous study that used a similar insert of ¾ length, albeit in a different shoe [12]. The implication of higher midfoot pressure requires further investigation but feasibly could influence how well the insert is tolerated over a longer period of wear. Contoured inserts are thought to distribute plantar pressures by increasing surface contact area; however, we did not observe significant differences in contact area between conditions.

Our findings suggest that impact force was influenced by shoe characteristics rather than the inserts used in this study. Peak magnitude of the vGRF was not different between shoe conditions, but average and maximum loading rate was lower when walking in the test shoe (Condition B) compared to the control shoe (Condition A), indicating an effect of the softer outsole material and greater heel‐toe pitch. No differences were observed between insert conditions within the test shoe. Although another study has reported altered loading rate when walking in different shoes in asymptomatic individuals [39], others have found no difference [26, 40] and most have solely reported peak magnitude [16, 17]. Comparison to studies is further limited by the variety of different shoe comparisons with little description of specific shoe characteristics [26, 39, 40]. Future studies should ensure that shoe design features and materials are clearly reported.

Our findings suggest that underfoot comfort also appeared to be influenced by shoe design rather than the insert. The same insert (flat low‐density ethylene vinyl acetate) was more comfortable when used in the test shoe (Condition B) compared to the control shoe (Condition A). Differences in comfort exceeded a clinically meaningful magnitude (> 10 mm on visual analogue scale) [33]; however, it is uncertain which aspect of shoe design specifically enhanced comfort. Lane et al. [20] reported that shoe sole hardness had no immediate effect on comfort scores across shoe conditions with identical heel‐toe pitch. It is possible that the increased heel‐toe pitch and reduced loading rate of vGRF demonstrated in our test shoe could help explain the immediate effect on comfort, particularly in a population with symptomatic PHP. We found no differences in comfort between insert conditions in the test shoe. Existing literature [28] suggests that softer insoles are perceived as more comfortable, and the influence of contouring is conflicting as to whether flat or contoured insoles are perceived as more comfortable. Although the inserts used in this study varied in contouring and material properties, the differences may not have been substantial enough to notice an immediate effect on comfort, whereas further testing of comfort over time would be warranted.

Although this study had several strengths, including concurrent evaluation of pressure‐relieving/force attenuation effect and comfort, investigation of a nonathletic ‘everyday’ shoe and blinded data analysis, the results should be interpreted in the context of some limitations. First, we report effects in a group of middle‐aged, obese (group mean age = 47 years, BMI = 30.7 kg/m^2^) women, and although this represents a common demographic in the presentation of PHP, results may not be generalisable to all individuals with PHP. Second, this study was partially funded by a women's footwear manufacturer, which is why women's footwear was investigated. Although a systematic review of studies in the pharmaceutical industry suggests that sponsored studies can lead to bias [41], the industry partner had limited input during preliminary study conception/design and was not involved in data collection or analysis. Third, the literature has identified many influences on comfort [28], which include, but are not limited to, structural and functional aspects of shoe design. It is possible that anatomical and physiological characteristics also influenced comfort scores and warrant further investigation. Fourth, this study investigated the immediate effects of various shoes and inserts, and investigation of effects over a longer period is warranted. Fifth, although participants were not informed of specific design features/differences, we were unable to conceal the external appearance of the shoe. Previous studies suggest that perceptual factors including cost, appearance and colour may influence comfort but are unlikely to affect plantar pressures and vGRF characteristics [28, 42]. Lastly, we heat moulded the Formthotics (Condition F) to fit the test shoe, but the devices were not individually customised, as would occur in a clinical setting.

## 5 Conclusion

The results of this study suggest that in women with PHP, both shoe and shoe insert characteristics are important considerations for reducing heel peak pressures. Shoe outsole design, rather than shoe insert, seemed to have the greatest effect on impact forces and comfort. Reducing mechanical load on structures of the heel during walking may be a mechanism by which shoes in combination with inserts reduce symptoms in PHP. Given that a person takes thousands of steps daily, the potential cumulative effect may be significant. Comfortable footwear is likely to improve adherence, which is an important consideration for both the clinic setting and the design of clinical trials. Our findings support expert recommendations regarding footwear selection for PHP and may help identify footwear for future clinical trials.

## Author Contributions

**Melinda M. Franettovich Smith:** conceptualization, formal analysis, investigation, methodology, project administration, visualisation, writing – original draft, Writing – review and editing. **Wolbert van den Hoorn:** methodology, formal analysis, software, writing – review and editing. **Adele van den Hoek:** formal analysis, writing – review and editing. **Graham Kerr:** methodology, resources, writing – review and editing. **Sheree E. Hurn:** conceptualization, formal analysis, funding acquisition, methodology, project administration, resources, supervision, Writing – review and editing.

## Ethics Statement

This study was approved by the Institutional Human Research Ethics Committee (#4889) and participants provided written informed consent.

## Conflicts of Interest

Sheree E. Hurn declares that prior to the commencement of this study, she was a consultant receiving funding (< $10k) from FRANKIE4 footwear. To manage this potential conflict of interest for the current research project, Melinda Smith was employed as an independent researcher through the government‐industry funding scheme. Other authors have no competing interests to declare.

## Data Availability

Because of the commercially sensitive nature of the research, research data are not shared.

## References

[1] P. J. Bennett , “Types of Foot Problems Seen by Australian Podiatrists,” Foot 22, no. 1 (2012): 40–45, 10.1016/j.foot.2011.11.002. 22265449

[2] H. B. Menz , K. P. Jordan , E. Roddy , and P. R. Croft , “Characteristics of Primary Care Consultations for Musculoskeletal Foot and Ankle Problems in the UK,” Rheumatology 49, no. 7 (2010): 1391–1398, 10.1093/rheumatology/keq092. 20403912 PMC2886311

[3] M. Cotchett , S. E. Munteanu , and K. B. Landorf , “Depression, Anxiety, and Stress in People With and Without Plantar Heel Pain,” Foot & Ankle International 37, no. 8 (2016): 816–821, 10.1177/1071100716646630. 27137796

[4] D. B. Irving , J. L. Cook , M. A. Young , and H. B. Menz , “Impact of Chronic Plantar Heel Pain on Health‐Related Quality of Life,” Journal of the American Podiatric Medical Association 98, no. 4 (2008): 283–289, 10.7547/0980283. 18685048

[5] K. B. Landorf , M. R. Kaminski , S. E. Munteanu , G. V. Zammit , and H. B. Menz , “Health‐Related Quality of Life Is Substantially Worse in Individuals With Plantar Heel Pain,” Scientific Reports 12, no. 1 (2022): 15652, 10.1038/s41598-022-19588-5. 36123358 PMC9485111

[6] C. Drake , G. A. Whittaker , M. R. Kaminski , et al., “Medical Imaging for Plantar Heel Pain: A Systematic Review and Meta‐Analysis,” Journal of Foot and Ankle Research 15, no. 1 (2022): 4, 10.1186/s13047-021-00507-2. 35065676 PMC8783477

[7] A. Phillips and S. McClinton , “Gait Deviations Associated With Plantar Heel Pain: A Systematic Review,” Clinical Biomechanics 42 (2017): 55–64, 10.1016/j.clinbiomech.2016.12.012. 28095359

[8] T. A. Koc Jr , C. G. Bise , C. Neville , D. Carreira , R. L. Martin , and C. M. McDonough , “Heel Pain – Plantar Fasciitis: Revision 2023: Clinical Practice Guidelines Linked to the International Classification of Functioning, Disability and Health From the Academy of Orthopaedic Physical Therapy and American Academy of Sports Physical Therapy of the American Physical Therapy Association,” Journal of Orthopaedic & Sports Physical Therapy 53, no. 12 (2023): CPG1–CPG39, 10.2519/jospt.2023.0303. 38037331

[9] D. L. Riddle , M. Pulisic , P. Pidcoe , and R. E. Johnson , “Risk Factors for Plantar Fasciitis: A Matched Case‐Control study,” Journal of Bone and Joint Surgery American Volume 85‐A, no. 5 (2003): 872–877.[Erratum Appears in J Bone Joint Surg Am. 2003 Jul;85‐A(7):1338]. 10.2106/00004623-200305000-00015 12728038

[10] R. A. Werner , N. Gell , A. Hartigan , N. Wiggerman , and W. M. Keyserling , “Risk Factors for Plantar Fasciitis Among Assembly Plant Workers,” Physical Medicine and Rehabilitation 2, no. 2 (2010): 110–116, 10.1016/j.pmrj.2009.11.012. 20193937

[11] D. Morrissey , M. Cotchett , A. Said J'Bari , et al., “Management of Plantar Heel Pain: A Best Practice Guide Informed by a Systematic Review, Expert Clinical Reasoning and Patient Values,” British Journal of Sports Medicine 55, no. 19 (2021): 1106–1118, bjsports‐2019‐101970, 10.1136/bjsports-2019-101970. 33785535 PMC8458083

[12] D. R. Bonanno , K. B. Landorf , and H. B. Menz , “Pressure‐Relieving Properties of Various Shoe Inserts in Older People With Plantar Heel Pain,” Gait & Posture 33, no. 3 (2011): 385–389, 10.1016/j.gaitpost.2010.12.009. 21256025

[13] B. Van Lunen , N. Cortes , T. Andrus , M. Walker , M. Pasquale , and J. Onate , “Immediate Effects of a Heel‐Pain Orthosis and an Augmented Low‐Dye Taping on Plantar Pressures and Pain in Subjects With Plantar Fasciitis,” Clinical Journal of Sport Medicine 21, no. 6 (2011): 474–479, 10.1097/jsm.0b013e3182340199. 22011796

[14] J. K. K. Chia , S. Suresh , A. Kuah , J. L. J. Ong , J. M. T. Phua , and A. L. Seah , “Comparative Trial of the Foot Pressure Patterns Between Corrective Orthotics, Formthotics, Bone Spur Pads and Flat Insoles in Patients With Chronic Plantar Fasciitis,” Annals Academy of Medicine Singapore 38, no. 10 (2009): 869–875, 10.47102/annals-acadmedsg.v38n10p869. 19890578

[15] W.‐L. Hsi , J.‐S. Lai , and P.‐Y. Yang , “In‐Shoe Pressure Measurements With a Viscoelastic Heel Orthosis,” Archives of Physical Medicine and Rehabilitation 80, no. 7 (1999): 805–810, 10.1016/s0003-9993(99)90231-9. 10414766

[16] A. Fong Yan , P. J. Sinclair , C. Hiller , C. Wegener , and R. M. Smith , “Impact Attenuation During Weight Bearing Activities in Barefoot vs. Shod Conditions: A Systematic Review,” Gait & Posture 38, no. 2 (2013): 175–186, 10.1016/j.gaitpost.2012.11.017. 23245643

[17] S. Franklin , M. J. Grey , N. Heneghan , L. Bowen , and F.‐X. Li , “Barefoot vs Common Footwear: A Systematic Review of the Kinematic, Kinetic and Muscle Activity Differences During Walking,” Gait & Posture 42, no. 3 (2015): 230–239, 10.1016/j.gaitpost.2015.05.019. 26220400

[18] M. I. V. Mientjes and M. Shorten , “Contoured Cushioning: Effects of Surface Compressibility and Curvature on Heel Pressure Distribution,” Footwear Science 3, no. 1 (2011): 23–32, 10.1080/19424280.2010.536587.

[19] J. Anderson , A. E. Williams , and C. Nester , “Development and Evaluation of a Dual Density Insole for People Standing for Long Periods of Time at Work,” Journal of Foot and Ankle Research 13, no. 1 (2020): 42, 10.1186/s13047-020-00402-2. 32641098 PMC7341629

[20] T. J. Lane , K. B. Landorf , D. R. Bonanno , A. Raspovic , and H. B. Menz , “Effects of Shoe Sole Hardness on Plantar Pressure and Comfort in Older People With Forefoot Pain,” Gait & Posture 39, no. 1 (2014): 247–251, 10.1016/j.gaitpost.2013.07.116. 23968972

[21] M. W. Creaby , K. May , and K. L. Bennell , “Insole Effects on Impact Loading During Walking,” Ergonomics (London) 54, no. 7 (2011): 665–671, 10.1080/00140139.2011.592600. 21770753

[22] A. Daryabor , T. Kobayashi , H. Saeedi , S. M. Lyons , N. Maeda , and S. S. Naimi , “Effect of 3D Printed Insoles for People With Flatfeet: A Systematic Review,” Assistive Technology 35, no. 2 (2023): 169–179, 10.1080/10400435.2022.2105438. 35882078

[23] C. D. Miller , E. R. Laskowski , and V. J. Suman , “Effect of Corrective Rearfoot Orthotic Devices on Ground Reaction Forces During Ambulation,” Mayo Clinic Proceedings 71, no. 8 (1996): 757–762, 10.4065/71.8.757. 8691896

[24] L. Yung‐Hui and H. Wei‐Hsien , “Effects of Shoe Inserts and Heel Height on Foot Pressure, Impact Force, and Perceived Comfort During Walking,” Applied Ergonomics 36, no. 3 (2005): 355–362, 10.1016/j.apergo.2004.11.001. 15854579

[25] X. Zhao , M. Wang , G. Fekete , J. S. Baker , H. Wiltshire , and Y. Gu , “Analyzing the Effect of an Arch Support Functional Insole on Walking and Jogging in Young, Healthy Females,” Technology and Health Care 29, no. 6 (2021): 1141–1151, 10.3233/thc-181373. 30452428

[26] A. J. Rawcliffe , S. M. Graham , R. J. Simpson , et al., “The Effects of British Army Footwear on Ground Reaction Force and Temporal Parameters of British Army Foot Drill,” Journal of Strength and Conditioning Research 34, no. 3 (2020): 754–762, 10.1519/jsc.0000000000002139. 28800005

[27] M. Ho , J. Nguyen , K. Talbot , et al., “Immediate Comfort Perception of 3D‐Printed Foot Orthoses in Individuals With Unilateral Heel Pain,” Prosthetics and Orthotics International 46, no. 1 (2022): 31–36, 10.1097/pxr.0000000000000068. 35179521 PMC8865620

[28] H. B. Menz and D. R. Bonanno , “Footwear Comfort: A Systematic Search and Narrative Synthesis of the Literature,” Journal of Foot and Ankle Research 14, no. 1 (2021): 63, 10.1186/s13047-021-00500-9. 34876192 PMC8650278

[29] A. Finestone , V. Novack , A. Farfel , A. Berg , H. Amir , and C. Milgrom , “A Prospective Study of the Effect of Foot Orthoses Composition and Fabrication on Comfort and the Incidence of Overuse Injuries,” Foot & Ankle International 25, no. 7 (2004): 462–466, 10.1177/107110070402500704. 15319103

[30] F. Faul , E. Erdfelder , A.‐G. Lang , and A. Buchner , “GPower 3: A Flexible Statistical Power Analysis Program for the Social, Behavioral, and Biomedical Sciences,” Behavior Research Methods 39, no. 2 (2007): 175–191, 10.3758/bf03193146. 17695343

[31] A. C. Redmond , J. Crosbie , and R. A. Ouvrier , “Development and Validation of a Novel Rating System for Scoring Standing Foot Posture: The Foot Posture Index,” Clinical Biomechanics 21, no. 1 (2006): 89–98, 10.1016/j.clinbiomech.2005.08.002. 16182419

[32] J. Dawson , H. Doll , J. Coffey , C. Jenkinson , B. F. Oxford , and Ankle Clinical Research G , “Responsiveness and Minimally Important Change for the Manchester‐Oxford Foot Questionnaire (MOXFQ) Compared With AOFAS and SF‐36 Assessments Following Surgery for Hallux Valgus,” Osteoarthritis and Cartilage 15, no. 8 (2007): 918–931, 10.1016/j.joca.2007.02.003. 17383907

[33] K. Mills , P. Blanch , and B. Vicenzino , “Identifying Clinically Meaningful Tools for Measuring Comfort Perception of Footwear,” Medicine & Science in Sports & Exercise 42, no. 10 (2010): 1966–1971, 10.1249/mss.0b013e3181dbacc8. 20216463

[34] A. B. Putti , G. P. Arnold , L. Cochrane , and R. J. Abboud , “The Pedar In‐Shoe System: Repeatability and Normal Pressure Values,” Gait & Posture 25, no. 3 (2007): 401–405, 10.1016/j.gaitpost.2006.05.010. 16828288

[35] V. Racic , A. Pavic , and J. M. Brownjohn , “Number of Successive Cycles Necessary to Achieve Stability of Selected Ground Reaction Force Variables During Continuous Jumping,” Journal of Sports Science and Medicine 8, no. 4 (2009): 639–647. 24149607 PMC3761548

[36] S. Schreven , P. J. Beek , and J. B. J. Smeets , “Optimising Filtering Parameters for a 3D Motion Analysis System,” Journal of Electromyography and Kinesiology 25, no. 5 (2015): 808–814, 10.1016/j.jelekin.2015.06.004. 26159504

[37] M. Pijnappels , M. F. Bobbert , and J. H. van Dieën , “Changes in Walking Pattern Caused by the Possibility of a Tripping Reaction,” Gait & Posture 14, no. 1 (2001): 11–18, 10.1016/s0966-6362(01)00110-2. 11378420

[38] J. P. Belinda Barton , Medical Statistics: A Guide to SPSS, Data Analysis and Critical Appraisal, 2nd ed. (Wiley, 2014).

[39] K.‐O. Yi , “The Effects of Shoe Type on Ground Reaction Force,” Korean Journal of Sport Biomechanics. 21, no. 1 (2011): 009–016, 10.5103/kjsb.2011.21.1.009.

[40] M. A. Lafortune and E. M. Hennig , “Cushioning Properties of Footwear During Walking: Accelerometer and Force Platform Measurements,” Clinical Biomechanics 7, no. 3 (1992): 181–184, 10.1016/0268-0033(92)90034-2. 23915727

[41] J. Lexchin , L. A. Bero , B. Djulbegovic , and O. Clark , “Pharmaceutical Industry Sponsorship and Research Outcome and Quality: Systematic Review,” British Medical Journal 326, no. 7400 (2003): 1167–1170. 12775614 10.1136/bmj.326.7400.1167 PMC156458

[42] S. T. McCAW , M. E. Heil , and J. Hamill , “The Effect of Comments About Shoe Construction on Impact Forces During Walking,” Medince and Science in Sports and Exercise 32, no. 7 (2000): 1258–1264, 10.1097/00005768-200007000-00012. 10912891

